# Manual acupuncture plus usual care versus usual care alone in the treatment of endometriosis-related chronic pelvic pain: study protocol for a randomised controlled feasibility study

**DOI:** 10.1186/s40814-017-0152-9

**Published:** 2017-07-06

**Authors:** Mike Armour, Caroline A. Smith, Siobhan Schabrun, Genevieve Z. Steiner, Xiaoshu Zhu, Kenny Lawson, Jing Song

**Affiliations:** 1 NICM, Western Sydney University, Campbelltown Campus, Sydney, Australia; 2 Brain Rehabilitation and Neuroplasticity unit, Western Sydney University, Campbelltown Campus, Sydney, Australia; 3 Centre for Health Research, Campbelltown Campus, Western Sydney University, Campbelltown Campus, Sydney, Australia; 4 Department of Obstetrics and Gynaecology, SWSLHD Camden and Campbelltown Hospitals, Sydney, Australia

**Keywords:** Acupuncture, Endometriosis, Pelvic pain, EEG, CPM, Inflammation, Protocol

## Abstract

**Background:**

Endometriosis is the most common cause of chronic pelvic pain worldwide. Non-surgical treatments are effective for only 30–50% of women and have a significant side effect burden that leads to high discontinuation rates. Surgery can be effective but is expensive and invasive, and symptoms tend to recur within 5 years. There is early evidence that acupuncture may be effective in treating endometriosis-related chronic pelvic pain, showing clinically significant analgesia. Both levels of inflammation and pain processing have been shown to be altered in women with chronic pelvic pain. Acupuncture has been shown to reduce inflammation and change central pain processing in other conditions, but research on women with endometriosis is currently lacking. The aim of this feasibility study is to provide data on recruitment rates, retention, appropriateness of outcome measures, minimal clinically important difference in numeric rated scales for pain and the potential effect of acupuncture on pain processing and markers of inflammation in endometriosis-related CPP.

**Methods:**

We will include women aged 18–45 years with a diagnosis of endometriosis via laparoscopy in the past 5 years. A total of 30 participants will be recruited and randomly allocated in a 1:1 ratio to receive acupuncture or usual care. Women in the acupuncture group will receive two 45-min treatment sessions per week for 8 weeks (total of 16 sessions). Women in the usual care group will continue with their current treatment regimen. The primary feasibility outcomes are recruitment rates, retention rates and the safety and acceptability of the intervention; secondary patient-centred outcomes include a change in 0–10 daily pelvic pain ratings, the Endometriosis Health Profile 30 (EHP-30) and changes in conditioned pain modulation, resting and task-related EEG activity and inflammatory markers. Analyses will be performed blind to group allocation.

**Discussion:**

This is a two-armed, assessor blind, randomised controlled feasibility trial. Data will be compared at baseline and trial completion 8 weeks later. Outcomes from this feasibility study will inform a larger, fully powered clinical trial should the treatment show trends for potential effectiveness.

**Trial registration:**

Australian New Zealand Clinical Trials Registry, ACTRN12617000053325 (http://www.ANZCTR.org.au/ACTRN12617000053325.aspx).

**Electronic supplementary material:**

The online version of this article (doi:10.1186/s40814-017-0152-9) contains supplementary material, which is available to authorized users.

## Background

Chronic pelvic pain (CPP) is pain in the pelvis of greater than 6-month duration that is severe enough to cause functional disability or require medical intervention [1]. Worldwide prevalence rates range between 5.7 and 26.6% [2]. Endometriosis is the presence of endometrial tissue outside the uterine cavity and is the most common cause of CPP [3] with 24 to 40% of women with CPP diagnosed with endometriosis [4, 5]. Endometriosis-related CPP includes a variety of pain symptoms including dysmenorrhea (period pain), dyspareunia (pain during sexual intercourse), dyschezia (pain on bowel motions) and dysuria (pain on urination). In addition to pelvic visceral or muscle pain [4], endometriosis reduces quality of life and increases absenteeism at work or school [6]. A recent cohort study of Australian women aged 34–39 years had a prevalence of confirmed endometriosis of 3.7% [7]. However, a 2005 cross-sectional survey of 1983 menstrual-aged Australian women found that 21.5% of women had experienced non-menstrual pelvic pain and 14.1% had experienced dyspareunia over the past 12 months [8]. This suggests that the prevalence of endometriosis may be significantly greater than 3.7%.

Chronic pain has been identified as a top five health concern by Australian women [9] and is a National Women’s Health priority [10]. Endometriosis impacts women’s health and wellbeing, including social activities [6], mental and emotional health [11], work and finances [6] and sexual relationships [12], and has been shown to reduce physical quality of life similar to that of cancer patients [6]. The economic cost burden of endometriosis is substantial [6, 13]. A European multi-country study found that, on average, the total economic cost burden was €9579 (2010 prices) comprising both health care costs (approx. 33%) and productivity losses (approx. 66%) due to absence from work as a result of pelvic pain [6]. Further, the productivity loss associated has been estimated to be similar to or higher than certain chronic disease burdens, including diabetes mellitus, Crohn’s disease and rheumatoid arthritis which are commonly recognised as priority disease areas across health sectors [14].

Current treatments such as non-steroidal anti-inflammatories, oral contraceptive pills and hormonal treatments have limited effectiveness [13], and the side effect profile is bothersome, with discontinuation rates of 25–50% [15]. Surgical interventions are costly and invasive, and recurrence rates for pain are high, with 50% of women having pain recurrence at 5 years post-surgery [3, 13]. Early evidence from two recent studies comparing verum (true) acupuncture to sham acupuncture have shown promise in reducing endometriosis-related CPP using a short course (10–16 sessions) of treatment [16, 17].

Acupuncture may reduce endometriosis-related CPP through multiple mechanisms including the potential to reduce prostaglandin expression [18], reduce nerve growth factor [19], increase endogenous opioid release [20] and reduce mechanical allodynia [19]. However, the effects of acupuncture on three key areas that are altered in women with chronic pelvic pain are still unclear: increased inflammatory markers, changes in neuronal functional connectivity, and altered endogenous pain modulation.

Interleukin-6 (IL-6) is a cytokine and inflammatory marker implicated in the pathogenesis of endometriosis [21]. Serum IL-6 levels raised in women with endometriosis [22, 23] are positively correlated with disease stage [24] and change in response to symptom severity during treatment [25]. Acupuncture has been shown to reduce IL-6 levels [18, 19], and one small study examined the effect of Japanese acupuncture on IL-6 in women with endometriosis, but due to recruitment issues, the study was too underpowered to detect any differences [17]; therefore, acupuncture’s effect on IL-6 in endometriosis remains unclear.

Neuroimaging research shows a correlation between endometriosis-related CPP, depression and anxiety and increased connectivity between the anterior insula and medial prefrontal cortex [26]. A recent systematic review shows acupuncture has the ability to regulate pain-related functional connectivity [27], but there has been no investigation of acupuncture’s effect on functional connectivity in women with CPP. Electroencephalography (EEG) is a simple, low-cost method that can be used to investigate functional connectivity [28]. Dysfunctional endogenous pain inhibition is also commonly found in chronic pain conditions [29]; however, its presence in endometriosis-related CPP has not yet been demonstrated.

Conditioned pain modulation (CPM) is a technique to investigate individual differences in pain inhibition in women with CPP and can be used to investigate changes in endogenous pain inhibition after acupuncture treatment. Investigation of these physiological biomarkers that could underpin the efficacy of acupuncture in conjunction with clinical outcomes has the potential to fill a significant gap in our knowledge of acupuncture’s potential mechanism(s) of action and is a key area for acupuncture research [30].

The aim of this study is to assess the feasibility and acceptability of acupuncture and to investigate any changes in physiological biomarkers when using acupuncture to treat endometriosis-related CPP. Primary feasibility objectives will be evaluated in terms of recruitment (interest to participate in the trial, identification of appropriate recruitment strategies, the appropriateness of eligibility criteria), retention (compliance with treatment attendance and dropout rates), safety (adverse event rates) and appropriateness of the outcome measures (compliance with data collection). Secondary patient-centred outcomes to be evaluated include a change in 0–10 daily pelvic pain ratings, the Endometriosis Health Profile 30 (EHP-30) and changes in conditioned pain modulation, resting and task-related EEG activity and inflammatory markers.

## Methods/design

This is an 8-week, two armed, parallel group assessor-blind, randomised controlled feasibility study.

Ethics approval for this study was granted in December 2016 by the Western Sydney University Human Research Ethics committee (H11984). The trial was registered with the Australian New Zealand Clinical Trials Registry (ACTRN12617000053325) on January 11, 2017. The study protocol (version 3, 6/2/17) (see Additional file 1) has been designed in accordance with the SPIRIT guidelines [31] (see Additional file 2), Good Clinical Practice guidelines (1996) and the STRICTA guidelines for reporting of acupuncture trials [32]. Any changes to the trial protocol will be communicated to all investigators, reflected in changes to the trial registry and will be first approved via the ethics committee.

### Recruitment, setting and informed consent

Recruitment will be primarily via Endometriosis Australia, a not-for-profit charity. Trial information and invitations to participate will be published on their website (http://www.endometriosisaustralia.org) as well as via their Facebook page and Twitter feed. A link to a screening page (http://nicm.edu.au/research/clinical_trials/endometriosis) will be provided by the recruiting organisation. Women will fill in a screening questionnaire, and their contact details will be collected. A researcher will contact potential participants, explain the study in detail and ensure the participant information sheet has been read and understood. Women who agree to participate will fill in a 4-week baseline pelvic pain diary that records the severity of both menstrual and non-cyclical pelvic pain. Four weeks is considered adequate to obtain baseline pain measures in women with endometriosis [33]. Potential participants will also need to provide a copy of the laparoscopic findings confirming a diagnosis of endometriosis. Upon returning these to the research team, eligibility will be confirmed and written consent obtained, including consent to take and store blood samples. The trial intervention will be conducted in Sydney, Australia, at both NICM at Western Sydney University’s Campbelltown campus, and several private acupuncture practices in the greater Sydney area.

### Eligibility criteria

Women will be eligible to participate in the trial if they:

- Are aged 18–45 years
- Have a laparoscopic diagnosis of endometriosis in the last 5 years as per current clinical guidelines [33–35]
- Are having regular menstruation (every 3–5 weeks)
- Have menstrual or non-menstrual pelvic pain rated ≥4/10 on a numeric rating scale
- Report at least one of the following: dysmenorrhea, dyspareunia, dyschezia or dysuria

Women will not be eligible to participate if they have had endometriosis surgery or started the oral or injectable contraceptive pill, GnRH-a or danazol, within the last 6 months. Women who do not meet the initial eligibility criteria will be logged. Women who decline to participate after initial contact will also be logged, with a reason for declining (if given).

For the EEG component of the study, women will be screened for suitability for EEG measurement. EEG exclusion criteria are as follows:

- Diagnosed psychiatric disorders including dissociative disorder, obsessive-compulsive disorder, personality disorder, schizophrenia and bipolar disorder
- History of drug and alcohol dependence or substance-related disorders
- History of seizures
- Head trauma with loss of consciousness
- Left-handedness

Participants who are not eligible for EEG measurement will still be accepted into the trial and will complete all other outcome measures.

### Randomization, allocation concealment and blinding

Practitioners and participants will not be blinded. Research assistants and others performing data collection, data entry and analysis will be blind to group allocation. Participants will be asked not to mention any details of their treatment or their group allocation to the assessor(s). If blinding is compromised, another assessor will be contacted and will complete the data collection. A computerised, internet-based central randomisation service (sealedenvelope.com) will be used to provide randomisation and allocation concealment. Women will be randomised in a 1:1 ratio between acupuncture and usual care. Once known, the primary investigator will communicate the group allocation to the participant.

### Treatment schedule

#### Acupuncture + usual care

Women in this group will receive 16 acupuncture treatments over 8 weeks, twice weekly with a minimum of 48 h between treatments, delivered by registered Chinese medicine practitioners in private clinics or by a registered Chinese medical practitioner at the clinic room at NICM. All study acupuncturists will have a minimum of 5 years experience, with a minimum of a bachelor’s level qualification in acupuncture, and will hold current Chinese Medicine practitioner registration with the Australian Health Practitioner Regulation Agency (AHPRA). Practitioners will be located in central and western Sydney areas. MA is also an acupuncturist and will train the practitioners in the trial protocol as well as deliver treatments to those women who find treatment at Campbelltown campus geographically convenient.

Acupuncture treatment will be delivered using a standardised fixed set of acupuncture points, based on those used in a previous pilot clinical trial using a traditional Chinese medicine (TCM) framework to treat endometriosis [16]. The set acupuncture points that will be delivered every session are SP6, SP8, SP10, ST29, ST36, CV3, CV4 and LR3. All participants will receive the same acupuncture points, with no individualisation based on TCM diagnosis. Table 1 outlines the rationale and anatomical location for these points based on the common presenting symptoms of endometriosis.

**Table 1:** Acupuncture point selection rationale

Acupuncture point	Relevant indication(s) for endometriosis [37]	Location
Sanyinjiao (SP6)	Irregular menstruation, uterine bleeding, dysmenorrhea, abdominal masses in women	3 *cun* ^a^ directly above the tip of the medial malleolus
Diji (SP8)	Irregular menstruation, dysmenorrhea, abdominal masses in women	On the medial aspect of the lower leg, 3 *cun* below SP9, on the line connecting the tip of the medial malleolus and SP9
Xuehai (SP10)	Irregular menstruation, dysmenorrhea, abdominal masses in women	When the knee is flexed, on the medial aspect of the thigh, the point is 2 *cun* above the mediosuperior border of the patella, on the bulge of the medial portion of m. quadriceps femoris.
Zhongji (CV3)	Masses below the umbilicus, severe pain below the umbilicus, abdominal masses, irregular menstruation, painful urination, infertility	On the anterior median line of the lower abdomen, 4 *cun* below the umbilicus
Guanyuan (CV4)	Fullness of the lower abdomen, back pain and twisting pain below the umbilicus that radiates to the genitals, painful urination, infertility	On the anterior midline, 3 *cun* below the umbilicus
Guilai (ST29)	Irregular menstruation, uterine masses, pain in the vagina, infertility	2 *cun* lateral to the anterior midline, level with CV 3
Zusanli (ST36)	Distension and pain of the abdomen	On the anterior aspect of the lower leg, 3 *cun* below ST 35, one finger breadth (middle finger) from the anterior crest of the tibia
Taichong (LR3)	Epigastric or abdominal pain, periumbilical pain, pain of the genitals, irregular menstruation, painful urinary dysfunction, difficult defecation	On the dorsum of the foot, in the depression distal to the junction of the first and second metatarsal bones

Acupuncture point nomenclature as per World Health Organisation guidelines for meridian alphabetic codes

^a^A *cun* is a measurement used in locating acupoints, and corresponds to the distance between the two medial ends of the creases of the interphalangeal joints, when the patient’s middle finger is flexed

Acupuncture points will be needled bilaterally, *DeQi* obtained and needles retained for 25–30 min. *DeQi* (the arrival of Qi) is the sensation generated by the insertion and/or manipulation of an acupuncture needle in an acupuncture point and is often described as a dullness, heaviness or distending sensation [36]. Point location and needling depth will be as specified in *A Manual of Acupuncture* [37]. Single-use, stainless steel needles of varying gauge (0.20 × 30.00 mm or 0.25 × 40.00 mm), dependent on body shape, will be used. All reporting of the acupuncture intervention in the final manuscript will conform to the STRICTA guidelines [32]. All participants in the acupuncture group will also continue with their usual care as currently prescribed or advised by either their general practitioner or gynaecological specialist, the specifics of which are the same as the usual care only group.

#### Usual care only

All participants will continue with their usual care as currently prescribed or advised by either their general practitioner or gynaecological specialist. Usual care will be based on specific medical advice that has been given to the participant but commonly involves the oral contraceptive pill or non-steroidal anti-inflammatory or analgesic medication. The use of usual care alone will allow the natural progression and fluctuations in CPP to be isolated from the effect of acupuncture.

### Prohibited and permitted concomitant treatment

No other TCM co-interventions (moxibustion, cupping or herbal medicine) will be permitted during the trial. Participants will continue with all prescribed medication and can use analgesics as needed for pain relief. All analgesic usage is noted in the daily pain diary.

### Assessment schedule

There is a 4-week screening period prior to trial entry, followed by an 8-week treatment intervention. Participants in both groups will need to attend baseline and end of trial measurements at NICM on Western Sydney University’s Campbelltown campus. Table 2 outlines the assessments during the total 12-week period.

**Table 2:** Timeline of treatment assessments and interventions

Period	Screening		Treatment								
Week		PB	B	1	2	3	4	5	6	7	8
Daily 0–10 pain diary	X			X	X	X	X	X	X	X	X
Presence of endometriosis comorbidities	X			X	X	X	X	X	X	X	X
Rescue analgesic medication usage	X			X	X	X	X	X	X	X	X
Proof of laparoscopic diagnosis	X										
Eligibility screening	X										
Informed consent signed		X									
Randomisation		X									
Demographic characteristics		X									
ENDOCOST Survey			X^a^								
EHP-30			X^a^								X
Internal Health Locus of Control measurement			X^a^								X
EXPECT questionnaire			X^a^								
CPM			X								X^b^
IL-6 levels			X								X^b^
EEG			X								X^b^
Acupuncture treatment				X	X	X	X	X	X	X	X
Safety assessment				X	X	X	X	X	X	X	X
CGI-I											X
Treatment satisfaction/feasibility measures											X

*PB* after screening questionnaire received and eligibility confirmed, *B* Baseline visit to NICM lab, *EHP-30* Endometriosis Health Profile 30-item questionnaire, *EEG* electroencephalography, *CPM* conditioned pain modulation test, *IL-6* interleukin 6, *CGI-I* Clinical Global Impression scale-Improvement

^a^Forms will be completed after randomisation and will be returned during the baseline visit

^b^Visit to NICM lab at end of week 8

### Outcome measures

Patient-reported outcomes, neurophysiological measures and blood samples will be collected at the various time points outlined in Table 2. Baseline demographic data on age, ethnicity, menstrual history, smoking, current medication and revised American Society of Reproductive Medicine (rASRM) classification [38] of endometriosis will be collected by the primary investigator (MA) prior to the baseline visit to NICM. Baseline 0–10 numeric rating scale (NRS) pain scores for both non-cyclical and menstrual pain will be collected prior to trial entry during the screening period. The EXPECT questionnaire will be used to determine expectancy and belief in acupuncture at baseline [39]. Financial and personal impact of endometriosis will be collected via the ENDOCOST questionnaire at baseline [40]. The traditional Chinese medicine differential diagnosis thought to underlie participants’ symptoms will be recorded by the treating acupuncturist. All pain scores and questionnaires are self-reported and all analyses will be performed blind to group allocation.

### Primary feasibility outcomes

The primary outcome for this trial is the feasibility and acceptability of the recruitment methods, intervention and outcome measures. Feasibility outcomes include the following:

- Recruitment rate (interest to participate in the trial, identification of appropriate recruitment strategies and the appropriateness of eligibility criteria)
  Recruitment rate will be calculated as the number of women who are eligible for randomisation out of those who express interest in the trial.
- Retention (compliance with treatment attendance and dropout rates)
  Retention will be calculated as the number of women who complete all 16 acupuncture sessions (in the acupuncture group) and both baseline and follow-up visits to the NICM lab (in both the acupuncture and usual care groups).
- Safety (adverse events)
  We will be collecting details of any adverse events that occur either during or after treatment in the acupuncture group.
- Appropriateness of the outcome measures (compliance with data collection)
  Appropriateness of the outcome measures will be measured by examining the missing data in the measures used.

### Secondary patient-centred outcomes

- The pelvic pain outcome measures are daily 0–10 NRS for non-cyclical pelvic pain and a separate daily 0–10 NRS used for menstrual pain during the period itself via a daily pain diary. Pain scores are to be recorded in the evening, with one number capturing the overall pain for the day. The 11-point numeric rating scale is recommended as being suitable to score both dysmenorrhea and pelvic pain in women with endometriosis [33]. Scores for menstrual (pain that occurs when menstrual bleeding is present) and non-menstrual pelvic pain will be considered separately, as summing them may obscure the true extent of potential improvement [33]. During the menstrual period, the single daily pain score will reflect menstrual-related pain, while outside of menses will reflect non-cyclical pain. Pain scores will be recorded daily for 4 weeks prior to trial entry to provide a suitable baseline and daily for 8 weeks during the trial period itself. Daily non-menstrual pain scores will be converted into a single non-menstrual pain scores at three time points, baseline, month 1 and month 2. This will be achieved by taking the mean of the daily pain scores for the previous 4 weeks, excluding any days where menstruation was noted. Menstrual pain scores will be calculated by the mean of the daily pain scores for each menstrual period at each of the three time points.
- The Endometriosis Health Profile 30 (EHP-30) is an endometriosis-specific patient-reported outcome that examines health-related quality of life (HRQoL) in women with endometriosis. The EHP-30 is a user-friendly, valid and reliable condition-specific tool to assess HRQoL [41] and contains questions on physical and emotional functioning as recommended by the IMMPACT guidelines [42]. The EHP-30 will be measured at baseline and at trial completion.
- The Clinical Global Impression scale (CGI). A modified version of the global improvement question [43] will be used at trial exit to determine the minimal clinically important difference in NRS rated pain scores in women with endometriosis, by using comparing changes in patients’ self-assessment scores with changes in NRS scores to derive a non-inferiority margin. Similar techniques have been used for VAS pain scores in women with endometriosis [43].
- Prevalence and severity of comorbidities. The daily prevalence and severity (mild, moderate or severe) of common endometriosis comorbidities including dyspareunia, dysuria and dyschezia.
- Analgesic medication usage. The use of any rescue analgesic medication (e.g. paracetamol or ibuprofen) will be logged using the daily pain diary.
- Internal health locus of control (iHLoC). Internal health locus of control is a measure of how much control individuals feel that they can exert over their own health, as opposed to their health being strongly influenced by either luck and/or chance or powerful external entities, such as God or a doctor [44]. Internal health locus of control will be measured at baseline and trial completion.
- Traditional Chinese medicine (TCM) acupuncture differential diagnosis. TCM texts emphasise the importance of treating both the root (the underlying cause) and the branch (symptomatic treatment) via accurate differential diagnosis [45]. The individualisation of treatment based on differential diagnosis is considered to be fundamental to acupuncture practice [46]. Collection of the TCM differential diagnosis given by the treating acupuncturist will provide data on any possible correlation between diagnosis at trial entry and other physiological biomarkers.

### Physiological biomarker collection and processing

- Human electroencephalography (EEG) is a non-invasive recording technique that captures ongoing electrical brain activity via strategically placed scalp electrodes. The excellent temporal resolution of EEG makes it the ideal methodological approach for investigating the neuronal mechanisms associated with intervention-related changes. EEG recordings will be undertaken between day 4 and 11 of the menstrual cycle for women not using oral contraceptives, or any day of the menstrual cycle where women are taking an active hormonal contraceptive treatment, to minimise hormonally related fluctuations that can influence recordings [26, 47]. Participants will have a 64-channel sintered Ag/AgCl electrode cap fitted. Electro-oculogram (EOG) will also be recorded using sintered Ag/AgCl electrodes placed 2 cm above and below the left eye for vertical movements and on the outer canthus of each eye for horizontal movements. Data will be acquired DC–70 Hz, digitised at 1000 Hz and amplified with a gain of 2816 using a Neuroscan Synamps2 digital signal-processing system and Neuroscan 4.5.1 Acquire software. A conducting gel will be inserted into all electrodes, and their impedances will be kept below 10 kΩ. Resting-state EEG activity (2 min each of eyes open, eyes closed and eyes open) will be recorded for 6 min; this will be used to quantify resting-state functional connectivity. A simple visual discrimination task (modified version of the AX-Continuous Performance Test (AX-CPT)) will then be presented to assess whether the intervention has affected the activated brain state. The modified AX-CPT allows the testing of sustained attention, processing speed and executive function (inhibition, error detection). Deficits in context-induced error processing from this version of the AX-CPT correlate well with performance on the Stroop task. Stimuli consisting of the letters A, X, B and Y will be presented in a quasi-random sequence. Participants are instructed to respond to the letter X (target) when it is proceeded by the letter A (cue). Stimuli are presented for a duration of 250 ms, at a fixed inter-stimulus interval (ISI) of 1.2 s. The probability of correct AX pairings will be *p* = .7 so that participants have a pre-potent response. Incorrect AY (cued nontarget; context-induced errors), BX (uncued target; context-free errors) and BY (uncued nontarget; random responding) pairings will each be presented at a probability of *p* = 1. A total of 500 stimuli will be presented (AX = 350, AY = 50, BX = 50 and BY = 50). Display and stimulus markers will be controlled by a separate stimulus computer using Compumedics Stim2 (4.0.09302005) software. Participants will be required to respond to correctly cued targets (AX) as quickly and as accurately as possible via a button press with the dominant hand using the Stim System Switch Response Pad (P/N 1141). Unrestricted temporal principal components analysis will be applied to quantify event-related potential (ERP) component amplitudes.
- Conditioned pain modulation (CPM). CPM is a well-established, reliable and safe measure of pain processing that is thought to indicate the function of descending pain control systems. This is examined as a change in the pain perceived in one body region (test stimulation) as a result of pain induced in another body region (conditioned stimulation). We will use pressure pain threshold (PPT) measurement as the test stimulation and cold pain (3 °C ice bath) as the conditioned stimulation. Three PPTs (test stimulation) will be measured before the application of cold pain (conditioned stimulation). Cold pain will be applied by placing the participants’ dominant hand (to the wrist) in a circulating water bath at a cold temperature (maximum 3 °C) for 2 min. The water temperature will be checked prior to each test to ensure that it is in accordance with the protocol. Three PPT measurements will be repeated 30 s after applying the conditioned stimulation. Participants will be asked to rate their pain during conditioned stimulation on a numeric rating scale (0–100) at 0 s, at 30 s and at the end of the trial. Pain scores will be maintained between 50 and 80/100 during testing.
- Interleukin-6 (IL-6) levels. A blood sample will be collected from participants by a trained phlebotomist during the first testing session (after informed consent and screening) and again at trial conclusion 8 weeks later. A trained phlebotomist will collect ~10 ml of blood (one vacutainer) from the median cubital vein (cubital fossa anterior to the elbow). Samples will be transported to the NICM pharmacology laboratory by a member of the research team for processing as per the Australian Standard “Packaging for surface transport of biological material that may cause disease in humans, animal and plants—AS4834”. All samples will be de-identified and stored in the NICM of −80 °C freezer following centrifugation. Samples will be thawed and batch tested at the conclusion of the trial. Serum samples will be tested in duplicate for IL-6 levels using Quantikine high sensitivity solid phase ELISA immunoassays (R&D Systems Human IL-6 Quantikine HS ELISA, In Vitro Technologies, Victoria, Australia).

### Criteria for success

This feasibility trial will be deemed a success and lead to the development of a proposal for a fully powered randomised controlled trial if:

- The acupuncture intervention is deemed to be safe and acceptable based on combination of participant satisfaction at the end of the trial, the type and rate of adverse events, and adherence rates (at least 75% of women completing at least 12 of the 16 treatment sessions).
- At least 30 participants are recruited during the 9-month trial recruitment period.
- At least 30% of those expressing interest in the trial are eligible to complete to the baseline measurements.
- At least 67% of participants complete the follow-up session at the NICM lab at the completion of the trial.

Modifications may be made to the protocol for the full study based on participant feedback and recruitment rates, including the addition of additional clinical/laboratory sites.

### Sample size

This trial is not designed to establish efficacy but rather to assess feasibility. At least 30 women in total will be recruited for this study which we estimate will answer our feasibility questions on recruitment and retention rates based on a previous feasibility study of acupuncture in Australian women of a similar age that recruited 20 women and had a dropout rate of 15% [48]. A minimum of fifteen women will be allocated to each group in a 1:1 ratio, with a predicted dropout rate of 10–15%.

### Statistical analysis

Baseline demographics will be reported using descriptive statistics. Primary feasibility outcome measures are descriptive statistics (measures of central tendency [e.g. mean], variability [e.g. standard deviation] and effect size [e.g. standardised mean difference and confidence intervals]) for recruitment rates, dropout rates, adverse event rates and acceptability data. An exploratory analysis of secondary patient-centred outcomes will also be undertaken. NRS levels for menstrual and non-menstrual pain will be analysed using repeated measures at baseline, month 1 and month 2 via a longitudinal linear mixed model analysis of variance with time and group as fixed effects and subject as a random effect. Changes in IL-6 levels and CPM scores will be assessed using paired *t* tests. Categorical data such as expectation levels and comorbidities will be analysed using Fisher’s exact tests. Missing data for the pain diaries will not be imputed as the linear mixed model analysis of variance does not require manual imputation. Missing data for other outcomes will be imputed using last observation carried forward (LOCF). Functional connectivity analyses will be applied to post-processed resting-state EEG data using eLORETA (exact low resolution brain electromagnetic tomography) [49–51]. Task-state post-processed EEG data (ERPs from the AX-CPT) will be submitted to an unrestricted principal components analysis with Varimax rotation to quantify ERP component (factor) amplitudes.

### Quality assurance

Quality assurance measures on needling technique will be carried out on practitioners during the study period by using an independent acupuncturist to check point selection and location at least once for each practitioner. Regular calibration and testing for CPM and EEG equipment will occur both prior to and during the data-recording period. The EEG laboratory has a SOP that all research assistants must adhere to when collecting data. The investigator GZS will oversee this process.

### Safety monitoring

At each treatment session, the treating acupuncturist will ask the participants if they have had any adverse events or reactions after the last treatment. Any adverse events or reactions that are thought to be causally associated with the intervention will be recorded in the practitioner’s log book and reported to the PI. Any adverse events reported to the PI will be discussed with the other investigators to determine clinical significance. All adverse events reported during the duration of the trial will be recorded under adverse events as part of the case report form.

## Discussion

Acupuncture is a low-risk treatment [52] that shows promise in targeting some of the physiological mechanisms underlying the pathogenesis of endometriosis, and preliminary clinical trials have shown clinically significant analgesia. Currently, there are no data on the minimal clinically important difference in pain when using the numeric rating scale in women with CPP. Understanding the minimal clinically important difference in pain is crucial for determining an appropriately powered clinical trial. In addition, the recruitment methods, safety and the acceptability of a twice weekly acupuncture intervention and the appropriateness of the three biomarkers as outcome measures are vital feasibility components that need to be understood prior to embarking on a fully powered randomised controlled trial. A fixed point selection was chosen as this has shown effectiveness in a previous clinical trial on TCM acupuncture for endometriosis [16]. This reduces the effect of treatment variation between practitioners but at the expense of ecological validity; however, as this is a feasibility study, this is not a significant issue. Previous trials for endometriosis have shown promising results after 10 treatments delivered twice per week [16] and after 16 treatments delivered twice per week [17]. In this study, we decided to treat twice weekly for 8 weeks (16 treatments) as this gives a greater cumulative dose of acupuncture delivered [53].

This study will be the first to investigate the possible mechanisms of acupuncture using two physiological techniques with this clinical population. No previous study has examined the role of the endogenous pain modulation system in women with endometriosis or used EEG to examine changes in functional connectivity after acupuncture treatment in women with chronic pelvic pain. This study addresses current methodological challenges and recommendations in acupuncture research [30], allowing a preliminary investigation into the correlation between clinical outcomes and changes in biomarkers and any relationship between biomarkers at entry and presenting acupuncture TCM differential diagnosis. An improved understanding of the possible mechanisms of action of acupuncture in altering pain processing and inflammation will guide future trial design to strengthen the evidence base for acupuncture.

### Trial status

Recruitment commenced February 2017.

## Additional files

Additional file 1: Treatment of endometriosis related chronic pelvic pain: study protocol v3. (DOCX 74 kb)

Additional file 2: SPIRIT 2013 Checklist: recommended items to address in a clinical trial protocol and related documents. (DOC 120 kb)

## Abbreviations

AHPRA: Australian Health Practitioner Regulation Agency
AUD: Australian dollars
CPM: Conditioned pain modulation
CPP: Chronic pelvic pain
EEG: Electroencephalogram
EHP-30: Endometriosis Health Profile 30
ERP: Event-related potential
IL-6: Interleukin-6
NRS: Numeric rating scale
SAR: Society for Acupuncture Research
SOP: Standard operating procedure
TCM: Traditional Chinese medicine
