# Supplementary material for: Manual acupuncture plus usual care versus usual care alone in the treatment of endometriosis-related chronic pelvic pain: study protocol for a randomised controlled feasibility study
Source: Pilot Feasibility Stud. 2017 Jul 6;4:10. doi: 10.1186/s40814-017-0152-9 (PMC5500924; doi:10.1186/s40814-017-0152-9)
Supplement: Supplementary file 1 — Treatment of endometriosis related chronic pelvic pain: study protocol v3. (DOCX 74 kb) [file 40814_2017_152_MOESM1_ESM.docx]

*Protocol*

**Treatment of endometriosis related chronic pelvic pain: a randomised controlled feasibility study of acupuncture.**

**Protocol Number** Version 3

**Date** 6th February 2017

**Grant:** ECR Development grant (Western Sydney University)

**Principal Investigators:**

Dr Mike Armour, PhD, The National Institute for Complementary Medicine, Western Sydney University.

Prof Caroline Smith, The National Institute for Complementary Medicine, Western Sydney University.

Assoc Prof Jason Abbott, Faculty of Medicine, University of New South Wales.

Dr Genevieve Steiner, The National Institute for Complementary Medicine, Western Sydney University.

Dr Siobhan Schabrun, Brain Rehabilitation and Neuroplasticity unit, Western Sydney University

Dr Xiaoshu Zhu, School of Science and Health, Western Sydney University.

A/Prof Kenny Lawson, Centre for Health Research, Western Sydney University.

**Study Centre:**

The National Institute for Complementary Medicine, Western Sydney University, Locked Bag 1797, Penrith, NSW 2751

# 1. Introduction

## 1.1 Background

Chronic pelvic pain (CPP) is pain of >6 months duration that is severe enough to cause functional disability or require medical or surgical treatment [1]. CPP includes a variety of pain symptoms including dysmenorrhea (pain during the menstrual period) , dyspareunia (pain on sexual intercourse), dyschezia (pain and difficulty with bowel movements) and dysuria (pain on urination), as well as pelvic visceral or muscle pain [2]. Worldwide prevalence rates are between 5.7% and 26.6% [3], affecting approximately 176 million women worldwide. CPP affects more than 70% of Australian women during their reproductive lifespan, most commonly with dysmenorrhea but 21.5% experience non-menstrual pelvic pain and 14.1% experience dyspareunia [4]. Endometriosis is the presence of endometrial tissue outside the uterus and the most common causes of CPP[5] with between 24% to 40% of women with CPP having diagnosed endometriosis [2, 6]. In addition to the CPP symptoms, 30-50% of women with endometriosis have impaired fertility [7]. A number of mechanisms are posited for the development of endometriosis[5], including over expression of PGE2, increases in nerve growth factor (NGF) and both central and peripheral sensitization (allodynia) [8]. Current treatments such as non-steroidal anti-inflammatories, oral contraceptive pills and hormonal treatments have limited efficacy [9] and the side effect profile is bothersome with discontinuation rates of 25-50% [10]. Surgical interventions are costly and invasive, and recurrence rates for pain are high, with 50% of women having pain recurrence at 5 years post-surgery [5, 9].

*Acupuncture*

Complementary and Alternative medicine (CAM) is commonly used by women in Australia with chronic pelvic pain, with an acupuncturist being the most common practitioner visited [11]. Acupuncturists in the community also reporting commonly seeing women with endometriosis [12]

There is little high quality evidence for using acupuncture in the treatment of endometriosis [13] however two recent studies have shown significant promise in reducing endometriosis related CPP using a short course (10-16 treatments) of acupuncture. One trial [14] found clinically significant reductions in VAS pain scores (-5.4 vs -1.2) and one trial [15] a reduction in a numerical rating scale (NRS) for pain (MD -1.4, 95% CI -2.6 to -0.2). Both trials compared acupuncture to different styles of sham acupuncture. However both trials suffer a number of methodological issues such as very small sample sizes [15], less common Japanese style of acupuncture [15] or the use of a cross-over design [14] which may not be appropriate in trials of acupuncture for cyclical pain issues. Acupuncture has multiple mechanisms by which it may reduce endometriosis related CPP including the potential to reduce PGE2 expression [16] reduce NGF [17], increase endogenous opioid release [18] and reduce mechanical allodynia [17].

*Neurological and biomarkers in Endometriosis related Chronic Pelvic Pain:*

Using neurological or biomarkers in conjunction with clinical outcomes has the potential to fill a significant gap in our current knowledge on acupuncture’s potential mechanism(s) of action and is a key area for acupuncture research according to a recent whitepaper written by the Society for Acupuncture Research (SAR) [19]. Interleukin-6 (IL-6) is a cytokine and inflammatory marker implicated in the pathogenesis of endometriosis[8]. Serum IL-6 levels are raised in women with endometriosis [20, 21], and, are positively correlated with disease stage [22] and change in response to symptom severity during treatment [23]. Acupuncture has been shown to reduce IL-6 levels [16, 17]. To date there has been one small study that has examined the effect of Japanese acupuncture vs sham acupuncture on IL-6 in women with endometriosis but due to severe recruitment issues the study was underpowered to detect any differences between groups [24]. Therefore acupuncture’s effect on IL-6 in endometriosis remains unclear.

Neuroimaging has shown a correlation between endometriosis related CPP, depression and anxiety, and increased connectivity between the anterior insula and medial prefrontal cortex [25]. A recent systematic review demonstrates acupuncture has the ability to regulate this pain-related functional connectivity [26] but no measurements of acupuncture’s effect on functional connectivity in women with CPP have been undertaken. Electroencephalography (EEG) is a simple, low cost way to investigate this connectivity[27]. Dysfunctional endogenous pain inhibition is also commonly found in chronic pain conditions [28] however its presence in endometriosis related CPP has not yet been demonstrated. Conditioned pain modulation (CPM) is a technique to investigate individual differences in pain inhibition in women with CPP. This study will explore changes in pain processing in women with endometriosis via changes in default network connectivity or endogenous pain modulation and the effect, if any, of acupuncture on these two possible mechanisms examined.

## 1.2 Study Rationale

Acupuncture is a low-risk treatment [29] and shows promise in treating some of the physiological mechanisms underlying the pathogenesis of endometriosis while preliminary clinical trials have shown clinically significant analgesia. There is only preliminary data on the feasibility of using IL-6, EEG and CPM as objective markers of changes in inflammation and central pain processing in women with CPP. In fact, we have no data on the clinically important difference in reducing pain from endometriosis to guide the design of an appropriately powered randomised controlled trial. These are vital components that need to be understood prior to embarking on a fully powered randomised controlled trial.

# 2. Study Objectives

The aim of this study is to (1) assess the feasibility and acceptability of acupuncture and (2) to investigate any changes in neurological or biomarkers when using acupuncture to treat endometriosis-related chronic pelvic pain.

The specific objectives are (1) To determine barriers to recruitment, appropriateness of eligibility criteria, acceptability and adherence to treatment in women with endometriosis related CPP. (2) To determine the minimal clinically important reduction in pain from endometriosis (3) To determine which, if any, objective markers of endometriosis CPP are altered by acupuncture (4) To determine if the endogenous pain modulation system is affected in women with CPP.

# 3. Study Design

A randomised controlled feasibility trial comparing acupuncture plus usual care to usual care alone for women with endometriosis related CPP.

# 4. Study Population

## 4.1 Sample Size

30 women in total, 15 participants per group.

## 4.2 Recruitment

We will recruit from the community using a patient support group Endometriosis Australia’s, Endoactive and The Pelvic Pain Foundation of Australia and social media portals. We will also recruit via Facebook advertisement for women aged 18-45 living within a 40km radius of Campbelltown, New South Wales, Australia.

## 4.3 Inclusion Criteria

- Women aged 18-45
- have a laparoscopic diagnosis of endometriosis in the last 5 years as per current clinical guidelines [30-32]
- having regular menstruation
- the presence of at least one of the following: dysmenorrhea, dyspareunia, dyschezia or dysuria rated >= 4/10 on a numeric rating scale.

## 4.4 Exclusion Criteria

## endometriosis surgery within the past 6 months,

## started oral or injectable contraceptive pill, GnRH-a or danazol within the last 6 months.

# 5. Participant selection and enrollment

## 5.1 Identifying participants

Recruitment will be primarily via Endometriosis Australia, Endoactive and Pelvic Pain foundation of Australia, all not for profit charities. Trial information and invitations to participate will be published on their respective websites as well as via their Facebook pages and twitter feeds. Recruitment will also occur via targeted Facebook advertising

5.2 Screening for eligibility:

For women expressing interest in the study they will make contact with the co-ordinating centre. Initial screening will be undertaken over the phone by the researcher. For those women that pass the initial screening, they will be invited to fill in a daily pain diary for four weeks to confirm eligibility and to provide baseline pain data.

To participate in the EEG sub-study women need to not meet any of the following criteria:

- Diagnosed psychiatric disorders including: dissociative disorder, obsessive-compulsive disorder, personality disorder, schizophrenia, bipolar disorder
- History of drug and alcohol dependence or substance-related disorders
- History of seizures
- Head trauma with loss of consciousness
- Left-handedness

If women meet any of these criteria they will not have EEG measurements performed but all other components of the study remain the same.

## 5.3 Consenting participants

For those that consent a trial entry form will record baseline clinical data as well as demographic characteristics. Randomisation will occur after baseline data has been collected.

## 5.4 Ineligible and non-recruited participants

Women who do not meet the initial eligibility criteria will be logged. Women who decline to participate after initial contact will also be logged, with a reason for declining (if given). Women who meet the trial inclusion criteria but cannot participate in the EEG sub-study will be logged with reasons for exclusion from the sub-study.

## 5.5 Randomisation

5.5.1 Randomisation

**Sequence generation**

The randomisation sequence will be created using the random number generator in SPSS by a researcher external to the research team. This researcher will hold the randomisation sequence.

**Allocation concealment**

PI Armour will call the central telephone number for treatment allocation once the participant has been enrolled in the study. Once known, PI Armour will communicate the group allocation to the participant.

5.5.2 Treatment allocation

Randomization will be 1:1 into one of two study groups:

Manual acupuncture + usual care:

Women in this group will receive 16 acupuncture treatments over 8 weeks (twice weekly – with a minimum of 2 days between treatments) in addition to their usual care, delivered by registered Chinese medicine practitioners in private clinics or at the clinic room (5.LG.07) at the National Institute for Complementary Medicine. Manual acupuncture will be delivered using a semi-standardized protocol based on previous research [14, 33].

Usual care:

Participants will continue with the usual care as currently prescribed or advised by either their general practitioner or gynecological specialist. Usual care will be based on specific medical advice that has been given to the participant, but commonly involves the oral contraceptive pill or non steroidal anti inflammatories or analgesic medication.

5.5.3: Withdrawal procedures

If a participant wishes to withdraw from the trial they will be immediately removed from treatment.

If a participant withdraws during intervention they will not be replaced however if they withdraw post randomization but prior to the first treatment they will be replaced.

# 6. Investigational therapy and controls

## 6.1 Acupuncture

A total of 16 treatments over 8 weeks will be administered (2/week). All study acupuncturists will be experienced, with a minimum of a Bachelors level qualification in Acupuncture and will hold current Chinese Medicine practitioner registration with AHPRA. Practitioners will be located in central and western Sydney areas. The PI (Mike Armour) is also an acupuncturist and will train the practitioners in the trial protocol as well as delivering treatments to those women who find treatment at Campbelltown campus geographically conveneient.

Acupuncture treatment will be delivered via a fixed set of acupuncture points, based on those used in a previous pilot clinical trial using a traditional Chinese medicine (TCM) framework to treat endometriosis [14]. Acupuncture points will include Spleen 6, Spleen 8, Spleen 10, Stomach 29, Stomach 36, Ren 3, Ren 4 and Liver 3.

Acupuncture points will be needled bilaterally and needles retained for 25-30 minutes. Point location and needling depth will be as specified in *A Manual of Acupuncture* [34]. Single use, stainless steel needles of varying gauge (.20 x 30mm or .25 x 40mm), dependent on body shape, will be used.

No other TCM co-interventions (moxabustion, cupping or herbal medicine) will be allowed during the trial.

## 6.2 Control group

Participants will continue with the usual care as currently prescribed or advised by either their general practitioner or gynecological specialist. The use of usual care alone will allow the natural progression and fluctuations in CPP to be isolated from the effect of acupuncture.

## 6.3 Prior and Concomitant Medications

### 6.3.1 Permitted medications

Subjects will continue with all prescribed medication and can use analgesics as needed for pain relief.

# 7. Study Assessments

## 7.1 Safety Assessments

At each treatment session the treating acupuncturist will ask the participants if they have had any adverse events or reactions after the last treatment. Any adverse events or reactions that are thought to be causally associated with the intervention will be recorded in the practitioners log book and reported to the primary investigator Mike Armour. Any adverse events reported to the PI will be discussed with the other investigators for clinical significance. All adverse events reported during the duration of the trial will be recorded under adverse events as part of the case report form.

Minor adverse events for acupuncture are expected to occur at a rate of around 8.6% however these were mostly minor with 2.2% requiring treatment [35]. The most common adverse events were minor bleeding or hematoma (6.1%) and pain (1.7%). Negligence or malpractice accounted for .1% of all adverse events and included broken or forgotten needles or pneumothorax. Bleeding and hematoma made up 58% of all adverse events. Neither of the two pneumothorax that occurred were life threatening. No deaths or permanent injuries were associated with the acupuncture treatments.

EEG is non-invasive research, so there is very minimal discomfort involved. It is possible that there may be a very small amount of discomfort when the electrode cap is fitted on participants' heads. The researcher will do everything possible to ensure that this experience is as comfortable for participants as possible. This will be done by ensuring that your cap is the correct size, and the researcher will check with participants throughout the experiment to ensure that the cap remains comfortable.

Participants will also be asked to provide a blood sample. The sample will be collected by a trained phlebotomist, and discomfort will be very minimal.

Participants will undergo a conditioned pain modulation test to measure the function of their descending pain systems. This test involves applying pressure stimuli to one part of the body while another part of the body is experiencing heat or cold pain. In this study we will be applying stimuli to the volar surface of the forearm. There are no risks associated with pressure stimuli. Some discomfort may be present when the pressure sensation first changes to one of pain. However, as this is a threshold test, the pressure stimulus will be ceased as soon as the participant notes that the sensation has changed from pressure to pain. There is a small risk of a burn with any application of heat. The risk of a burn will be reduced by screening and excluding participants with reduced peripheral sensation. In the unlikely event of a burn, standard operating procedure for treatment of a burn will be implemented. All staff will be familiar with these procedures.

The EEG, conditioned pain modulation and blood samples will all be taken during a single session at the same location at the National Institute of Complementary Medicine prior to receiving any trial intervention. This will be repeated at the conclusion of the trial intervention, or after 8 weeks in women who are allocated to usual care.

## 7.2 Study assessments

Key outcomes will be assessed at baseline and at the end of the intervention. Drawing of blood (IL-6) and neurological investigations (EEG and CPM) will be undertaken. Pain outcomes will be based on daily numeric rating scale (NRS) for non-cyclical pelvic pain and a separate NRS for menstrual pain. The Clinical Global Improvement (CGI) scale and The Endometriosis Health Profile (EHP-30) [36] will be used as secondary quality of life outcomes. Financial and personal impact of endometriosis will be collected via the ENDOCOST questionnaire [37]. Data will also be collected on safety and expectation (using the EXPECT questionnaire).Daily data on rescue medication use, presence and severity of other endometriosis symptoms (dyspareunia, dyschizia, dysuria) will be collected on the daily pain diary. Feasibility outcomes will include an assessment of interest to participate in the trial, identification of appropriate recruitment strategies, the appropriateness of eligibility criteria, compliance with treatment attendance and data collection, and dropout rates.

# 8. Data Collection

Baseline demographic data on age, ethnicity, menstrual history, smoking, current medication and revised American Society of Reproductive Medicine (rASRM) classification [38] of endometriosis will be collected by the primary investigator (Mike Armour) at prior to the first acupuncture session. Baseline pain scores for both non-cyclical and menstrual pain will be collected prior to trial entry. The EXPECT questionnaire will be used to determine expectancy and belief in acupuncture.

*Primary outcome measure:*

The primary outcome measures are daily 0-10 numeric rating scale (NRS) for non-cyclical pelvic pain and a separate daily 0-10 NRS used for menstrual pain during the period itself.

*Secondary outcome measures:*

The Clinical Global Improvement (CGI) scale and The Endometriosis Health Profile (EHP-30) [36] will be used as secondary quality of life outcomes. Data will also be collected on safety, expectation, rescue medication use, presence and severity of other endometriosis symptoms (dyspareunia, dyschizia, dysuria) as well as the financial and personal impact (e.g time off work, additional help required at home) of endometriosis related symptoms. Feasibility outcomes will include an assessment of interest to participate in the trial, identification of appropriate recruitment strategies, the appropriateness of eligibility criteria, compliance with treatment attendance and data collection, and dropout rates. components of the consultation and treatment sessions were important.

*Quality assurance:*

Quality assurance measures on needling technique will be carried out on practitioners during the study period by using an independent acupuncturist to check point selection and location at least once for each practitioner.

*Biomarkers:*

Drawing of blood (IL-6) will be undertaken by PI Steiner at baseline and at completion and neurological investigations (EEG and CPM) will be undertaken at baseline and again at the conclusion of treatment by PI Steiner and PI Schabrun.

Human electroencephalography (EEG) is a non-invasive recording technique that captures ongoing electrical brain activity via strategically placed scalp electrodes. The excellent temporal resolution of EEG makes it the ideal methodological approach for investigating the neural mechanisms associated with intervention-related changes. Participants will have an electrode cap fitted and resting-state EEG activity recorded for 10 minutes. A simple auditory discrimination task will then be presented to assess whether the intervention has affected the activated brain state.

Conditioned pain modulation (CPM): CPM is a well-established, reliable and safe measure of pain processing that is thought to indicate the function of descending pain control systems. This is examined as a change in the pain perceived in one body region (test stimulation) as a result of pain induced in another body region (conditioned stimulation). We will use PPT measurement as the test stimulation and heat pain (1°C above HPT) or cold pain (3°C ice bath) as the conditioned stimulation using the CPM System (Thermal Sensory Analyzer, TSA-2001, Q-Sense-CPM, Medoc Ltd, Ramat Yishai, Israel). Three PPTs (test stimulation) will be measured before the application of heat or cold pain (conditioned stimulation). Either heat or cold pain will be used. The heat pain will be applied via a 30 x 30 mm thermode on the volar surface of the forearm. Cold pain will be applied via placing the subjects dominant hand (to the wrist) in a circulating water bath at a cold temperature (maximum 3 °C) for 2 minutes. The water temperature will be checked prior to each test to ensure that it was in accordance with the protocol. Three PPT measurements will be repeated 30 seconds after applying the conditioned stimulation. Participants will be asked to rate their pain during conditioned stimulation on a numeric rating scale (0-100) at 0 s, 30 s and at the end of the trial. Pain scores will be maintained between 50 and 80/100 during testing.

# 9. Statistics and Data Analysis

## 9.1 Sample Size calculation:

There is no sample size calculation undertaken for this study due to its nature as a feasibility study. A previous feasibility study on acupuncture [39] has shown that a sample size of 30 is sufficient to provide data on feasibility outcomes.

## 9.2 Proposed Analysis:

Baseline demographics will be reported using descriptive statistics along with recruitment rates and other feasibility data. NRS levels for pain and CPM will be analysed using a mixed model ANCOVA. Changes in IL-6 levels will be assessed using a paired t-test. Categorical data such as expectation will be analysed using Fishers exact.

# 10. Monitoring and quality assurance

## 10.1 Project management and trial management group

The primary investigator, Mike Armour, will have regular meetings with the other investigators and will discuss recruitment, compliance, retention and follow up as well as the implementation of the overall trial protocol. These meetings will also discuss any adverse event data reported by study acupuncturists.

Mike Armour will also have regular email/phone conversations with other study acupuncturists to discuss any issues with recruitment, compliance, retention and follow up as well as the implementation of the overall trial protocol. In addition to this quality control will be undertaken at several points during the trial with each study acupuncturist being assessed at least once.

There is no data monitoring committee for this study.

# 11. Good Clinical Practice Module

## 11.1 Ethical conduct of the study

The research has been submitted to the Western Sydney University Human Research Ethics Committee.

## 11.2 Investigator responsibilities

11.2.1 Informed consent

All participants will be required to give informed consent prior to being randomized. Informed consent can be given electronically or via post.

11.2.2 Emergency contact with Investigators

All participants will be given emergency contact numbers of both the study acupuncturists and the primary investigator Mike Armour

11.2.3 Investigator indemnification

Following ethical approval, clinical trial insurance will be registered with the University

11.2.4 Study site staff

Mike Armour

11.2.5 Data Recording

All data will be entered electronically into a secure database with offsite backups at the National Institute for Complementary Medicine, Western Sydney University.

11.2.6 Confidentiality

All data is confidential. All study participants will be allocated a Study ID

11.2.7 Data protection

All electronic files will be password protected. All paper files will be kept in locked filing cabinets at Western Sydney University.

# 12. Reporting, Publication and Notification of Results

## 12.1 Authorship policy

The team of primary investigators will be the authors of the manuscript.

## 12.2 Publication

The study results will be presented at appropriate conferences and published in relevant journals.

# 13. References

1. Howard F, Perry P, Carter J, El-Minawi A. Pelvic Pain: diagnosis and management. Philadelphia: Lippincott Williams and Wilkins; 2000.

2. Whitaker LH, Reid J, Choa A, McFee S, Seretny M, Wilson J et al. An Exploratory Study into Objective and Reported Characteristics of Neuropathic Pain in Women with Chronic Pelvic Pain. PLoS One. 2016;11(4):e0151950. doi:10.1371/journal.pone.0151950.

3. Ahangari A. Prevalence of chronic pelvic pain among women: an updated review. Pain Physician. 2014;17(2):E141-7.

4. Pitts MK, Ferris JA, Smith AM, Shelley JM, Richters J. Prevalence and correlates of three types of pelvic pain in a nationally representative sample of Australian women. Med J Aust. 2008;189(3):138-43.

5. Hickey M, Ballard K, Farquhar C. Endometriosis. BMJ. 2014;348:g1752. doi:10.1136/bmj.g1752.

6. Mowers EL, Lim CS, Skinner B, Mahnert N, Kamdar N, Morgan DM et al. Prevalence of Endometriosis During Abdominal or Laparoscopic Hysterectomy for Chronic Pelvic Pain. Obstet Gynecol. 2016;127(6):1045-53. doi:10.1097/AOG.0000000000001422.

7. Macer ML, Taylor HS. Endometriosis and infertility: a review of the pathogenesis and treatment of endometriosis-associated infertility. Obstet Gynecol Clin North Am. 2012;39(4):535-49. doi:10.1016/j.ogc.2012.10.002.

8. Lundeberg T, Lund I. Is there a role for acupuncture in endometriosis pain, or 'endometrialgia'? Acupunct Med. 2008;26(2):94-110.

9. Brown J, Farquhar C. Endometriosis: an overview of Cochrane Reviews. Cochrane Database Syst Rev. 2014(3):CD009590. doi:10.1002/14651858.CD009590.pub2.

10. Sinaii N, Cleary SD, Younes N, Ballweg ML, Stratton P. Treatment utilization for endometriosis symptoms: a cross-sectional survey study of lifetime experience. Fertil Steril. 2007;87(6):1277-86. doi:10.1016/j.fertnstert.2006.11.051.

11. Fisher C, Adams J, Hickman L, Sibbritt D. The use of complementary and alternative medicine by 7427 Australian women with cyclic perimenstrual pain and discomfort: a cross-sectional study. BMC Complement Altern Med. 2016;16:129. doi:10.1186/s12906-016-1119-8.

12. Smith CA, Armour M, Betts D. Treatment of women's reproductive health conditions by australian and new zealand acupuncturists. Complement Ther Med. 2014;22(4):710-8. doi:10.1016/j.ctim.2014.06.001.

13. Zhu X, Hamilton KD, McNicol ED. Acupuncture for pain in endometriosis. Cochrane Database Syst Rev. 2011;9:CD007864. doi:10.1002/14651858.CD007864.pub2.

14. Rubi-Klein K, Kucera-Sliutz E, Nissel H, Bijak M, Stockenhuber D, Fink M et al. Is acupuncture in addition to conventional medicine effective as pain treatment for endometriosis? A randomised controlled cross-over trial. Eur J Obstet Gynecol Reprod Biol. 2010;153(1):90-3. doi:10.1016/j.ejogrb.2010.06.023.

15. Wayne PM, Kerr CE, Schnyer RN, Legedza ATR, Savetsky-German J, Shields MH et al. Japanese-style acupuncture for endometriosis-related pelvic pain in adolescents and young women: results of a randomized sham-controlled trial. Journal of pediatric and adolescent gynecology. 2008;21(5):247-57.

16. McDonald JL, Cripps AW, Smith PK. Mediators, Receptors, and Signalling Pathways in the Anti-Inflammatory and Antihyperalgesic Effects of Acupuncture. Evid Based Complement Alternat Med. 2015;2015:975632. doi:10.1155/2015/975632.

17. McDonald JL, Cripps AW, Smith PK, Smith CA, Xue CC, Golianu B. The anti-inflammatory effects of acupuncture and their relevance to allergic rhinitis: a narrative review and proposed model. Evid Based Complement Alternat Med. 2013;2013:591796. doi:10.1155/2013/591796.

18. Mayor D. An exploratory review of the electroacupuncture literature: clinical applications and endorphin mechanisms. Acupunct Med. 2013;31(4):409-15. doi:10.1136/acupmed-2013-010324.

19. Langevin HM, Wayne PM, Macpherson H, Schnyer R, Milley RM, Napadow V et al. Paradoxes in acupuncture research: strategies for moving forward. Evid Based Complement Alternat Med. 2011;2011:180805. doi:10.1155/2011/180805.

20. Martinez S, Garrido N, Coperias JL, Pardo F, Desco J, Garcia-Velasco JA et al. Serum interleukin-6 levels are elevated in women with minimal-mild endometriosis. Hum Reprod. 2007;22(3):836-42. doi:10.1093/humrep/del419.

21. Bedaiwy MA, Falcone T, Sharma RK, Goldberg JM, Attaran M, Nelson DR et al. Prediction of endometriosis with serum and peritoneal fluid markers: a prospective controlled trial. Hum Reprod. 2002;17(2):426-31.

22. Mosbah A, Nabiel Y, Khashaba E. Interleukin-6, intracellular adhesion molecule-1, and glycodelin A levels in serum and peritoneal fluid as biomarkers for endometriosis. Int J Gynaecol Obstet. 2016;134(3):247-51. doi:10.1016/j.ijgo.2016.01.018.

23. Iwabe T, Harada T, Sakamoto Y, Iba Y, Horie S, Mitsunari M et al. Gonadotropin-releasing hormone agonist treatment reduced serum interleukin-6 concentrations in patients with ovarian endometriomas. Fertil Steril. 2003;80(2):300-4.

24. Wayne PM, Kerr CE, Schnyer RN, Legedza ATR, Savetsky-German J, Shields MH et al. Japanese-style acupuncture for endometriosis-related pelvic pain in adolescents and young women: results of a randomized sham-controlled trial. J Pediatr Adolesc Gynecol. 2008;21(5):247-57. doi:10.1016/j.jpag.2007.07.008.

25. As-Sanie S, Kim J, Schmidt-Wilcke T, Sundgren PC, Clauw DJ, Napadow V et al. Functional Connectivity is Associated With Altered Brain Chemistry in Women With Endometriosis-Associated Chronic Pelvic Pain. J Pain. 2016;17(1):1-13. doi:10.1016/j.jpain.2015.09.008.

26. Villarreal Santiago M, Tumilty S, Macznik A, Mani R. Does Acupuncture Alter Pain-related Functional Connectivity of the Central Nervous System? A Systematic Review. J Acupunct Meridian Stud. 2016;9(4):167-77. doi:10.1016/j.jams.2015.11.038.

27. Sockeel S, Schwartz D, Pelegrini-Issac M, Benali H. Large-Scale Functional Networks Identified from Resting-State EEG Using Spatial ICA. PLoS One. 2016;11(1):e0146845. doi:10.1371/journal.pone.0146845.

28. Tracey I, Mantyh PW. The cerebral signature for pain perception and its modulation. Neuron. 2007;55(3):377-91. doi:10.1016/j.neuron.2007.07.012.

29. Macpherson H, Scullion A, Thomas KJ, Walters S. Patient reports of adverse events associated with acupuncture treatment: a prospective national survey. Qual Saf Health Care. 2004;13(5):349-55. doi:10.1136/qhc.13.5.349.

30. Vincent K, Kennedy S, Stratton P. Pain scoring in endometriosis: entry criteria and outcome measures for clinical trials. Report from the Art and Science of Endometriosis meeting. Fertil Steril. 2010;93(1):62-7. doi:10.1016/j.fertnstert.2008.09.056.

31. Practice Committee of the American Society for Reproductive Medicine. Treatment of pelvic pain associated with endometriosis: a committee opinion. Fertil Steril. 2014;101(4):927-35. doi:10.1016/j.fertnstert.2014.02.012.

32. Kennedy S, Bergqvist A, Chapron C, D'Hooghe T, Dunselman G, Greb R et al. ESHRE guideline for the diagnosis and treatment of endometriosis. Hum Reprod. 2005;20(10):2698-704. doi:10.1093/humrep/dei135.

33. Armour M. The effectiveness of acupuncture in the treatment of primary dysmenorrhea : a mixed methods study: Western Sydney University; 2015.

34. Deadman P, Al-Khafaji M, Baker K. A manual of acupuncture. 2nd, revised ed. Hove, East Sussex, England: Journal of Chinese Medicine; 1998.

35. Witt CM, Pach D, Brinkhaus B, Wruck K, Tag B, Mank S et al. Safety of acupuncture: results of a prospective observational study with 229,230 patients and introduction of a medical information and consent form. Forsch Komplementmed. 2009;16(2):91-7. doi:10.1159/000209315.

36. Khong SY, Lam A, Luscombe G. Is the 30-item Endometriosis Health Profile (EHP-30) suitable as a self-report health status instrument for clinical trials? Fertil Steril. 2010;94(5):1928-32. doi:10.1016/j.fertnstert.2010.01.047.

37. Simoens S, Hummelshoj L, Dunselman G, Brandes I, Dirksen C, D'Hooghe T et al. Endometriosis cost assessment (the EndoCost study): a cost-of-illness study protocol. Gynecol Obstet Invest. 2011;71(3):170-6. doi:10.1159/000316055.

38. Revised American Society for Reproductive Medicine classification of endometriosis: 1996. Fertil Steril. 1997;67(5):817-21.

39. Smith CA, Pirotta M, Kilbreath S. A feasibility study to examine the role of acupuncture to reduce symptoms of lymphoedema after breast cancer: a randomised controlled trial. Acupunct Med. 2014;32(5):387-93. doi:10.1136/acupmed-2014-010593.
